# Chemerin Impairs In Vitro Testosterone Production, Sperm Motility, and Fertility in Chicken: Possible Involvement of Its Receptor CMKLR1

**DOI:** 10.3390/cells9071599

**Published:** 2020-07-01

**Authors:** Anthony Estienne, Maxime Reverchon, Agnieszka Partyka, Guillaume Bourdon, Jérémy Grandhaye, Alix Barbe, Erika Caldas-Silveira, Christelle Rame, Wojciech Niżański, Pascal Froment, Joelle Dupont

**Affiliations:** 1 INRA UMR85 Physiologie de la Reproduction et des Comportements, France CNRS UMR7247 Physiologie de la Reproduction et des Comportements, France Université François Rabelais de Tours F-37041 Tours, IFCE F-37380 Nouzilly, France; anthony.estienne@inrae.fr (A.E.); guillaume.bourdon@inrae.fr (G.B.); jeremy.grandhaye@inrae.fr (J.G.); alix.barbe@inrae.fr (A.B.); Erika.Caldas-Silveira@inrae.fr (E.C.-S.); christelle.rame@inrae.fr (C.R.); pascal.froment@inrae.fr (P.F.); 2 SYSAAF—Syndicat des Sélectionneurs Avicoles et Aquacoles Français, Centre INRA Val de Loire, F-37380 Nouzilly, France; maxime.revercho@inrae.fr; 3 Department of Reproduction and Clinic of Farm Animals, Wrocław University of Environmental and Life Sciences, 50-375 Wrocław, Poland; agnieszka.partyka@up.wroc.pl (A.P.); wojciech.nizanski@upwr.edu.pl (W.N.)

**Keywords:** chemerin, sperm, chicken, spermatozoa motility, steroidogenesis, testis

## Abstract

The chemokine chemerin is a novel adipokine involved in the regulation of energy metabolism but also female reproductive functions in mammals. Its effects on male fertility are less studied. Here, we investigated the involvement of chemerin in chicken male reproduction. Indeed, the improvement of the sperm of roosters is a challenge for the breeders since the sperm quantity and quality have largely decreased for several years. By using specific chicken antibodies, here we show that chemerin and its main receptor CMKLR1 (chemokine-like receptor 1) are expressed within the chicken testis with the lowest expression in adults as compared to the embryo or postnatal stages. Chemerin and CMKLR1 are present in all testicular cells, including Leydig, Sertoli, and germinal cells. Using in vitro testis explants, we observed that recombinant chicken chemerin through CMKLR1 inhibits hCG (human chorionic gonadotropin) stimulated testosterone production and this was associated to lower 3βHSD (3beta-hydroxysteroid dehydrogenase) and StAR (steroidogenic acute regulatory protein) expression and MAPK ERK2 (Mitogen-Activated Protein Kinase Extracellular signal-regulated kinase 2) phosphorylation. Furthermore, we demonstrate that chemerin in seminal plasma is lower than in blood plasma, but it is negatively correlated with the percentage of motility and the spermatozoa concentration in vivo in roosters. In vitro, we show that recombinant chicken chemerin reduces sperm mass and individual motility in roosters, and this effect is abolished when sperm is pre-incubated with an anti-CMKLR1 antibody. Moreover, we demonstrate that fresh chicken sperm treated with chemerin and used for artificial insemination (AI) in hen presented a lower efficiency in terms of eggs fertility for the four first days after AI. Taken together, seminal chemerin levels are negatively associated with the rooster fertility, and chemerin produced locally by the testis or male tract could negatively affect in vivo sperm quality and testosterone production through CMKLR1.

## 1. Introduction

Some evidence indicates that dysregulation of the expression and/or secretion of hormones, mainly produced by adipose tissue called adipokines, is involved in the regulation of fertility [1,2,3]. Among adipokines, the newly characterized chemokine chemerin is suggested to influence the male reproductive tract [2,4]. Chemerin is a 16 kDa adipokine (also known as tazarotene-induced gene 2 (TIG-2) or retinoic acid receptor responder 2 (RARRES2) that was identified by [5]. It is secreted as an inactive precursor, pro-chemerin (18 kDa), and it is activated through post-translational carboxyl-terminal processing by a variety of proteinases [6]. Chemerin exerts its main biological functions through binding to its G protein-coupled receptor chemokine-like receptor 1 (CMKLR1), in humans also termed as chemerin receptor 23 (ChemR23) [7,8]. Besides their expression in adipose tissue, chemerin and CMKLR1 are expressed in various other tissues and cell populations [7]. A sex dimorphic pattern of chemerin expression, with higher levels in males compared to females, has been reported [9].

In males, RARRES2 and CMKLR1 genes are also expressed within the reproductive tract and more particularly in rat and human Leydig cells [10]. As described in females, chemerin inhibits in vitro gonadal steroidogenesis [11]. Indeed, chemerin reduces human chorionic gonadotropin (hCG)-induced testosterone production from rat primary Leydig cells, and this is associated with an inhibition of 3beta-hydroxysteroid dehydrogenase (3beta-HSD) and Mitogen-Activated Protein Kinase Extracellular signal-regulated kinase 1/2 (MAPK Erk1/2) signaling pathway [10,11]. Some evidence shows that chemerin is able to modulate various signaling pathways such as MAPK ERK1/2 [12] and P38 [13], Akt [14], and AMPK [14] in different mammalian cell types. These signaling pathways are involved in testosterone production [15,16,17,18]. However, in vivo, CMKLR1 deficiency in mice impairs the size of Leydig cells and their testosterone biosynthesis [19]. Thus, the molecular mechanism underlying the effect of CMKLR1 in inhibiting testosterone synthesis remains to be determined. In men, chemerin is in lower concentrations in seminal plasma compared to blood plasma [20,21]. However, seminal plasma chemerin concentrations are negatively correlated with sperm motility and they are not associated with BMI (Body Mass Index) [21]. Still, in humans, a study demonstrated that chemerin levels in the blood are lower in sub-fertile men compared to controls and are negatively correlated with plasma luteinizing hormone (LH), estradiol, and SHBG concentrations [22]. Thus, it is crucial to understand the role of the chemerin system in male fertility.

In the poultry breeding and production industry, artificial insemination technology is extensively employed to achieve higher reproductive efficiency [23]. In a breeder flock, a single male is responsible for fertilizing dozens of female birds. Thus, the fertility of the male is one of the first limiting factors for achieving the highest hatchability possible [24]. For several decades, the decrease in male chicken fertility has been associated with a decline in testicular weight, sperm production, and testosterone levels in animals [25]. To date, there are no data available about the chemerin role in male reproductive function in birds. Due to the huge agro-economical interest of chickens, ameliorating the reproduction of this species is a real challenge. In light of what it is known in human and rodent sperm function, studying the chemerin role on sperm function in chicken has a real sense in terms of fundamental understanding and agronomical applications. Our hypothesis is that chicken chemerin may regulate male gonadal steroidogenesis like rat chemerin but also sperm quality and consequently, in vivo fertility. Thus, we investigated the molecular mechanisms of chemerin involved in testosterone production and sperm quality by using in vitro and in vivo experiments in chicken.

## 2. Materials and Methods

### 2.1. Ethical Issues

All experimental procedures were performed in accordance with the French National Guidelines for the care and use of animals for research purposes (certificate of authorization to experiment on living animals APAFIS number 10237-201706151202940v3), Ministry of Agriculture and Fish Products, and a favorable notice of ethics committee of Val de Loire N°19.

### 2.2. Animals

All animals (rooster and hen) and chicken embryo (Cobb 500, from Hendrix Genetics (Saint Laurent de la Plaine, France)) were reared at “Pôle Expérimental Avicole de Tours” (INRA, Nouzilly, France) according to the traditional conditions of breeding. They were killed by electrical stunning and bled out as recommended by the ethical committee. To study chemerin and CMKLR1 expression in chicken testes during development, 70 roosters were studied at different stages: Embryo day 17, hatching, postnatal day 5, 10, 26, 42, and week 40 (*n* = 10 animals per stage). To analyze the in vitro effects of recombinant chicken chemerin on testosterone production, 120 10-day-old male chicks were used to perform testes explants. Testes were collected after slaughtering and kept in a cold saline buffer during the dissection. Fifty 40-week-old roosters were used to study the effect of recombinant chicken chemerin motility (mass and individually). Finally, 20 roosters and 32 hens (all 27-weeks-old) were used to test the effects of recombinant chicken chemerin on the egg fertilization.

### 2.3. Biological Samples

Blood samples from 36 adult roosters were collected from the occipital sinus into heparin tubes, and plasma was recovered after centrifugation (5000× *g* for 10 min at 4 °C) and then stored at −20 °C until use. Sperm was collected on the same 36 adult roosters at week 40 by manual stimulation. Sperm samples were centrifuged (5000× *g* for 10 min at 4 °C), and seminal plasma was stored at −20 °C for further investigations. Testis samples were obtained at different ages by dissection after animal slaughtering. Some testes samples at day 10 have been used for in vitro explant culture, and others samples have been stored at −80 °C for RT-qPCR and Western-blot.

### 2.4. Antibodies and Chicken Chemerin Recombinant Protein

Rabbit polyclonal antibodies to phospho-AMPKα (Thr172), phospho-MAPK ERK1/2 (Thr202/Tyr204), phospho-MAPK P38 (Thr180/Tyr182), total AKT, MAPK ERK1/2, and MAPK P38 were purchased from New England Biolabs Inc. (Beverly, MA, USA). Rabbit polyclonal antibodies to phospho-AKT (Ser 473) were purchased from Santa Cruz Biotechnology (Santa Cruz, CA, USA). Rabbit polyclonal antibodies to AMPKα1/2 were obtained from Upstate Biotechnology Inc. (Lake, Placid, NY, USA). Mouse monoclonal antibody to vinculin was obtained from Sigma. All antibodies were used at 1/1000 dilution in Western blotting. The recombinant chicken chemerin protein (full length, rRARRES2) was obtained from the Gallus Gallus sequence (NM_001277476.1), produced in Ecoli and purified by chromatography column-based on His-Tag in denaturation conditions (Agro-Bio, La Ferté Saint Aubin, France). The choice of chemerin concentrations used in the different assays (50, 150, and 500 ng/mL) was based on the chicken plasma chemerin concentrations (between 150 and 250 ng/mL) that we determined in different protocols [26,27]. Thus, we chose a lower concentration (50 ng/mL) and higher concentration (500 ng/mL) than that observed in plasma. We also tested these chicken chemerin concentrations on primary chicken Sertoli and granulosa cells, and we observed a significant effect of chicken chemerin at 150 ng/mL on the phagocytosis (Sertoli cells) and progesterone secretion (granulosa cells) (Manuscript in revision). Monoclonal chicken chemerin antibodies were produced, and their specificity was tested as previously described by [28].

### 2.5. Production of Antibodies against Chicken CMKLR1

Specific antibodies against chicken CMKLR1 were produced by AgroBio (Orleans, France). Briefly, 2 peptides corresponding to 20 amino-terminal residues (DDSDTYDYLDYTYEEPGSV, Chem20) and 18 carboxy-terminal residues (HRSFSKMSSMTEKETTVL, Chem18) of chicken chemerin were conjugated to keyhole limpet hemocyanin using sulfhydryl chemistry (Sigma Genosys, Woodlands, TX, USA) (cf Figure S1). One hundred fifty micrograms of both conjugated Chem20 and Chem18 were emulsified with an equal volume of complete Freund’s adjuvant and injected into 2 New Zealand White rabbits. Secondary immunizations were performed at day 14, 34, 57, 77, and 98 (relative to primary immunization) followed by bleeding on day 105. Blood samples were collected from an ear vein for titer testing in microtiter plates at day 42, 63, and 84. The specific humoral immune response to targeted antigens (Chem 20 and Chem 18) was evaluated by a direct ELISA using 96-well microtiter plates. The IgG from the antiserums was purified by protein G-affinity chromatography with the use of a HiTrap protein-G column (GE Healthcare, Parcay-Meslay, France), and the protein content was quantified with the BCA kit (Sigma-Aldrich, l’Isle d’Abeau Chesnes). We demonstrated the specificity of the antiserum by Western blot showing a 42 kDa signal in chicken liver, and muscle (pectoralis major) lysates. This 42 kDa signal was eliminated when immunoblotting was performed in the presence of pre-immune rabbit serum (Figure S1).

### 2.6. Chicken Chemerin ELISA Assay

Both blood and seminal plasma concentrations of chemerin (*n* = 36 animals) were obtained using chicken-specific kit E112C0104 (sensitivity 1 pg/mL) (Hölzel Diagnostika, Koln, Germany). The measurements were carried out according to the manufacturer’s protocol with intra- and inter-assay coefficients of variation < 6%. The absorbance was measured at 450 nm and then compared with reference values.

### 2.7. Testis Explants In Vitro Culture

Testes from 120 males chicks 10-day-old have been collected by dissection after slaughtering and kept in saline solution for about 30 min at room temperature -before use. For each culture, 20 testes have been pooled together and have been finely cut in small pieces of 1 mm of diameter. The equivalent of 2 testes was put in culture in each well of one 96 wells culture plate and incubated with various concentrations of recombinant chicken chemerin (Rec Chicken Chem, 0, 50, 200, and 500 ng for 48 h at 37 °C in the presence or absence of hCG (2 UI/mL, Sigma-Aldrich, l’Isle d’Abeau Chesnes) in DMEM culture medium (Sigma-Aldrich, l’Isle d’Abeau Chesnes) without serum. To determine whether the observed effects were mediated by CMKLR1, we blocked chicken chemerin/CMKLR1 signaling with a specific homemade rabbit anti-chicken CMKLR1 antibody (chicken CMKLR1 Ab, 10 μg/mL) beginning 1 h prior to application of the Rec Chicken Chem in the presence or absence of hCG and persisting throughout 48 h of stimulation. Conditioned culture medium was kept at −20 °C before use for hormonal assays. Testes explants have been kept at −80 °C before their use for RT-qPCR and Western Blot experiments. Six independent cultures have been performed (1 culture each week for 6 weeks).

### 2.8. mRNA Expression of RARRES2 and Its Receptor, CMKLR1 in Testes and mRNA Expression of 3βHSD, p450SCC, and STAR in In Vitro Testis Explants

Total RNA was extracted from the total testes from embryonic day 17 (E17) to 40-week-old roosters (10 animals per stage) and from in vitro testis explants by homogenization of 100 mg of tissue in the lysis buffer reagent of a total RNA extraction kit according to the manufacturer’s recommendations (NucleoSpin RNA, Macherey-Nagel, Hoerdt, France). The cDNA was generated by reverse transcription (RT) of total RNA (1 μg) in a mixture comprising 0.5 mM of each deoxyribonucleotide triphosphate (dATP, dGTP, dCTP and dTTP), 2 M of RT buffer, 15 μg/μL of oligodT, 0.125 U of ribonuclease inhibitor, and 0.05 U of Moloney murine leukemia virus reverse transcriptase (MMLV) for one hour at 37 °C. Real-time PCR was performed using the MyiQ Cycle device (Bio-Rad, Marnes-la-Coquette, France), in a mixture containing SYBR Green Supermix 1X reagent (Bio-Rad, Marnes la Coquette, France), 250 nM specific chicken primers (Invitrogen by Life Technologies, Villebon sur Yvette, France, chemerin forward 5′-CGCGTGGTGAAGGATGTG-3′, chemerin reverse 5′-CGACTGCTCCCTAAAGAGGAACT-3′; CMKLR1 forward 5′-CGGTCAACGCCATTTGGT-3′ CMKLR1 reverse 5′-GGGTAGGAAGATGTTGAAGGAA-3′; 3βHSD forward 5′-TACTGCTGGAAGAAGATGAG-3′ 3βHSD reverse 5′-CAAGGTGTCAATGATGGAAG-3′; p450SCC forward 5′-TGAATATCATCAGCCCCCGC-3′ p450SCC reverse 5′-GTAGGGCTTGTTGCGGTAGT-3′; STAR forward 5′-TGCCATCTCCTACCAACA-3′ STAR reverse 5′-CATCTCCATCTCGCTGAAG-3′;EEF1 alpha forward 5′-AGCAGACTTTGTGACCTTGCC-3′, and EEF1 alpha reverse 5′-TGACATGAGACAGACGGTTGC-3′) and 5 μL of cDNA (diluted 5-fold) for a total volume of 20 μL. The samples were duplicated on the same plate and the following PCR procedure used: After an incubation of 2 min at 50 °C and a denaturation step of 10 min at 95 °C, samples were subjected to 40 cycles (30 s at 95 °C, 30 s at 60 °C and 30 s at 72 °C). The levels of expression of messenger RNA were standardized to one reference gene (*EEF1*). For each gene (*chemerin*, *CMKLR1*, *3βHSD*, *p450SCC*, and *STAR*), expression was calculated according to primer efficiency (E) and quantification cycle (Cq), where expression = E^−Cq^. Then, the relative expression of the target gene to the *EEF1* alpha reference gene was analyzed.

### 2.9. Western Blot

Testes collected from embryonic day 17 (E17) to 40-week-old rooster (10 animals per stage) and testes in vitro explants were lysed using an Ultraturax (Invitrogen™ by Life Technologies™, Villebon sur Yvette, France) in lysis buffer (Tris 1 M (pH 7.4), NaCl 0.15 M, EDTA 1.3 mM, EGTA 1 mM, VO43−23 mM, NaF 0.1 M, NH2PO41%, Triton 0.5%). The lysates were centrifuged for 20 min at 17,000 g at 4 °C and the supernatant containing proteins was collected and kept on ice. The protein concentration of testes lysates and plasma (blood and seminal) was measured using the bicinchoninic acid (BCA) protein assay (Interchim, Montluçon, France). Tissue lysate protein (60 μg), blood and seminal plasma (5 μg) were mixed with Laemmli buffer 5 X and proteins were denatured for 5 min by heat shock at 95 °C. Proteins were loaded in an electrophoresis sodium dodecyl sulfate-polyacrylamide gel (12% for high protein weight (110–20 kDa) and 15% for low protein weight (<20 kDa)). Then, proteins were transferred to a nitrocellulose membrane. Membranes were blocked with Tris-Buffered Saline Tween buffer containing 0.05% of Tween 20 and 5% of milk for 30 min at room temperature. Membranes were incubated overnight at 4 °C with the appropriate primary antibody. Then, membranes were incubated 90 min at room temperature with a Horse Radish Peroxidase-conjugated anti-rabbit or anti-mouse IgG. Proteins of interest were detected by enhanced chemiluminescence (Western Lightning Plus-ECL, Perkin Elmer, Villebon-sur-Yvette, France) with a G-box SynGene (Ozyme, St Quentin en Yvelines, France) and GeneSnap software (Ozyme, St Quentin en Yvelines, France). Then, proteins were quantified with GeneTools software. The results were expressed as the intensity signal in arbitrary units after normalization.

### 2.10. Immunofluorescence

Fresh spermatozoa were fixed with PAF 4% for 15 min at room temperature and were deposited on slide. To saturate the aldehyde groups, the slides were incubated with PBS 1X/0.1 M glycine for 15 min at room temperature. Cells were permeabilized with 0.1% Triton X-100 (*w*/*v*) in PBS for 15 min, and nonspecific binding sites were blocked in 2% BSA for 15 min. Cells were incubated overnight at 4 °C with the following primary antibodies: Monoclonal chicken chemerin and polyclonal rabbit against chicken CMKLR1. Mouse or rabbit IgG purchased in (Sigma-Aldrich, l’Isle d’Abeau Chesnes, France) were used as a negative control. All primary antibodies were diluted at 1:100 in 1% BSA/PBS. After the first antibody incubation, spermatozoa were washed 3 times in PBS. Then, spermatozoa were incubated for 45 min at room temperature with a goat anti-rabbit or anti-mouse IgG Alexa Fluor^®^ 488 antibodies (diluted at 1:500 in 1% BSA/PBS). Cells were counterstained with 4′,6′-diamidino-2-phenylindole (DAPI), mounted on glass slides with Fluoroshield mounting medium (Sigma-Aldrich, l’Isle d’Abeau Chesnes, France) and examined using standard immunofluorescence microscopy. The analysis was performed on 100 spermatozoa in 3 different animals. Negative controls were performed by replacing antibody with mouse or rabbit IgG purchased in Sigma (Sigma-Aldrich, l’Isle d’Abeau Chesnes, France). In addition, as a negative control, we pre-incubated chicken chemerin antibody with chicken recombinant chemerin.

For testicular sections immunostained against chemerin, CMKLR1, CYP17, AMH, and VASA, testes from 10-day-old and adult (40-week-old) chicken were fixed in formalin, paraffin-embedded, and sectioned (7 µm) as described in [29]. Sections were dewaxed and rehydrated in xylene in decreasing concentrations of alcohol (100, 90, and 75%). Antigen retrieval was performed by steaming the sections in a microwave in citrate buffer (0.01 M), pH 6.0, for 5 min, then cooling for 20 min after 2 5-min washes in PBS. Sections were incubated at 4 °C overnight with the rabbit anti-AMH antiserum, rabbit anti-VASA antiserum, rabbit anti-CYP17 antibodies, and rabbit anti-chicken CMKLR1 diluted 1:200 in PBS containing 5% BSA (PBS-BSA). The monoclonal anti-chicken chemerin was also diluted 1:200 in PBS-BSA. The anti-AMH rabbit serum, produced by immunization with purified *N*-terminal His-tagged partial chicken AMH protein (from amino acid 66 to the *C* terminus), was a kind gift of Drs. D. Carré-Eusèbe and E. Oréal (INSERM U782, Clamart, France). The anti-chicken VASA rabbit serum, produced by immunization with purified *N*-terminal partial chicken CVH protein, was provided by Dr. B. Pain (U846 INSERM, Stem Cell, and Brain Research Institute, Lyon, Paris). The anti-CYP17 antibodies were purchased from Abcam (Paris, France). After washes in PBS–0.05% Tween 20 and PBS, slides were incubated in goat anti-rabbit or anti-mouse Alexa 488 (diluted at 1:500 in PBS for 1 h). After 3–5-min washes in PBS, sections were mounted with Vectashield. Negative controls were performed by replacing antibody with mouse or rabbit IgG purchased in (Sigma-Aldrich, l’Isle d’Abeau Chesnes, France). For each protein, immunostaining was performed on 5 testicular sections from 5 different animals.

### 2.11. Testosterone ELISA Assay

Testosterone concentrations in the conditioned culture media of testis explants were determined using commercial ELISA assays from Cayman Chemicals following the manufacturer instructions. The sensitivity of this assay was 0.01 ng/mL. The intra-assay and inter-assay coefficients of variation (CV) for each assay averaged <10%.

### 2.12. Evaluation of Sperm Motility Using IVOS (Integrated Visual Optical System)

Semen from 36 individual adult roosters (40-week-old) has been collected and treated individually with chicken recombinant chemerin at 50, 150, and 500 ng for 5 or 20 min at 37 °C with/without a pre-incubation for 1 hour at 4 °C with an anti-CMKLR1 antibody at 10 μg/mL. Computer-assisted sperm assessment (CASA) was performed by using a Hamilton-Thorne motility analyzer (Hamilton-Thorne Biosciences, Beverly, MA, USA) to determine total motility and kinematic characteristics of sperm movement. For each sample, sperm rapid and progressive velocity parameters were measured as indicators of sperm movement. The IVOS settings used were negative phase-contrast optics, recording rate of 60 frames per second, minimum contrast of 50, minimum cell size of 4 pixels, cell size gate of 25 pixels, and a cell intensity of 80. Three microliters of sperm from each sample were placed into a pre-warmed (37 °C) 100 μm standard counting chamber (Makler^®^ Counting Chamber, Clinisciences, Nanterre, France) before immediate transfer to IVOS. Sperm motility analysis was based on the 4–5 consecutive digitalized images obtained from a single field of view obtained using a 10× negative-phase contrast objective. Images were taken with a time-lapse of 1 s, and objects incorrectly identified as sperm were removed from the analysis. Motility parameters were evaluated as follows: Percentage of motile sperm (MOT), VCL (curvilinear velocity, 226 in μm/s), VSL (straight line velocity in μm/s), and VAP (average path velocity, in μm/s). Parameter means were calculated by the average of summary values obtained from each sample. For each sample of sperm of 36 animals, 1000 spermatozoa were analyzed at 37 °C in 100 μm standard counting chambers (Makler^®^ Counting Chamber, Clinisciences, Nanterre, France).

### 2.13. Sperm Membrane Integrity

Sperm membrane integrity was assessed with dual fluorescent probes, SYBR-14 and propidium iodide (PI) (Live/Dead Sperm Viability Kit, InvitrogenTM, Eugene, OR, USA) as described by [30]. The sperm samples were diluted with a commercial diluent provided in the kit to a concentration of 50 × 10^6^ spermatozoa per mL. Portions (300 μL) of the diluted samples were pipetted into specific tubes for cytometry, and 5 μL of SYBR-14 working solution was added. The working solution was obtained by diluting a commercial solution of SYBR-14 in distilled water at the ratio of 1:49. Samples were mixed and incubated at the room temperature for 10 min and then the cells were counterstained with 5 μL PI 5 min before analysis by flow cytometry. The PI negative and SYBR-14 positive population showing green fluorescence was considered alive, with the sperm plasma membrane intact. The experiment was performed at 2 times (5 and 20 min) after different treatments (chemerin 50 ng/mL, chemerin 150 ng chemerin 500 ng/mL, chicken CMKLR1 Ab (10 mg and chicken CMKLR1 Ab (10 mg/mL) + chemerin 500 ng/mL). It was repeated 6 times using different batches of adult chicken semen.

### 2.14. Acrosome Integrity

Acrosomal damage was assessed using phycoerythryn-labelled PNA (PE-PNA; Lectin from Arachis hypogaea, Merck Biosciences, Darmstadt, Germany). 10 μL of PE-PNA working solution (1 μg/mL) was added to 500 μL of diluted semen samples (50 × 10^6^ spermatozoa per mL) and incubated for 5 min at the room temperature in the dark. Following incubation, the supernatant was removed by centrifugation (500× *g* for 3 min), and the sperm pellets were re-suspended in 500 μL of the commercial buffer. Five μL of PI was added to samples before flow cytometry analysis. The experiment was performed at two times (5 and 20 min) after different treatments (chemerin 50 ng/mL, chemerin 150 ng/mL, chemerin 500 ng/mL, chicken CMKLR1 Ab (10 mg/mL) and chicken CMKLR1 Ab (10 mg/mL) + chemerin 500 ng/mL). It was repeated 6 times using different batches of adult chicken semen.

### 2.15. Determination of Ca^2+^ Levels in Spermatozoa

Control or treated sperm suspensions (20 μL; final concentration 2 × 10^6^ cells/mL) were centrifuged at 150 g for 15 min and lysed in RIPA buffer at 4 °C for 30 min, followed by sonication for 60 s on ice. The lysate was centrifuged at 10,000 g for 15 min. The concentration of Ca^2+^ was estimated in the supernatants using Arsenazo III (Sigma–Aldrich, Saint Quentin Fallavier, France) according to the modified method by [31]. The intensity of the purple complex formed with the reagent was read at 600 nm in a spectrophotometer (Labtech LT-4000MS; Labtech International Ltd., Uckfield, UK) with Manta PC analysis software. The protein concentration was estimated in the pellet by modified Lowry’s method [32]. The Ca^2+^ levels were calculated as μg/mL. The experiment was performed at 2 times (5 and 20 min) after different treatments (chemerin 50 ng/mL, chemerin 150 ng/mL, chemerin 500 ng/mL, chicken CMKLR1 Ab (10 mg/mL) and chicken CMKLR1 Ab (10 mg/mL) + chemerin 500 ng/mL). It was repeated 6 times (about 2 times per week) using different batches of adult chicken semen.

### 2.16. Mass Sperm Motility Assessment

We determined the effect of various concentrations of recombinant chicken chemerin (0, 50, 150, and 500 ng/mL) on the mass motility of rooster semen after 1, 3, 4, 10, and 20 min of treatment. To investigate the role of CMKLR1 in the chemerin effect on sperm mass motility, we pre-incubated the sperm samples with a specific homemade rabbit anti-chicken CMKLR1 antibody (chicken CMKLR1 Ab, 10 μg/mL) beginning 30 min prior to application of Rec Chicken Chem. The mass sperm motility was assessed as described in sheep [33] and chicken species [34]. Briefly, a drop of 5 μL of rooster semen collected on 12 different males and treated as previously described was deposited on a pre-warmed glass slide (≈37 °C), and the edge of the drop was observed at low magnification (objective 10×) on the thermally controlled stage of a phase-contrast microscope. Observations at the edge of the semen drop provided an assessment of the type and intensity of sperm waves and swirls movements, which was termed as wave motion or mass sperm motility. This mass sperm motility was scored subjectively from 0 (no motion) to 8 (numerous rapid waves) on a scale with steps equal to 1. In each experiment, we analyzed the effect of different concentrations of recombinant chicken chemerin (0, 50, 150, and 500 ng/mL) for various times of treatment (1, 3, 4, 10, and 20 min) in the absence and in the presence of the anti-chicken CMKLR1 antibody. This experiment was repeated 6 times (about 2 times per week) using 6 different semen samples. One semen sample was a pool of sperm from 12 different adult chicken males.

### 2.17. Artificial Insemination

The semen of 20 roosters (Cobb500) was collected and pooled to form a single sample. The concentration of spermatozoa of this pool was determined and then divided into 2 for control and recombinant chicken chemerin groups, respectively. The pool of sperm for the chemerin group was treated with 500 ng/mL of recombinant chicken chemerin for 30 min on ice, and the control group was treated with a vehicle under the same conditions. Sixteen hens in the control group and 16 hens in the sperm treated with chemerin group were artificially inseminated with 2 × 10^8^ spermatozoa from the 2 pools, respectively, on the 1st and 2nd day of the 27th week. Eggs from each hen were collected and counted daily for 3 weeks and incubated every 7 days (*n* = 200 eggs in total per group). The number of fertilized eggs was evaluated by candling after 7 days and 14 days of incubation. After candling at 7 days, eggs without clear viable embryos were opened to determine whether they contained an early dead embryo or an unfertilized oocyte. No early and late embryonic mortality was observed. Only clear eggs (unfertilized oocytes) were reported.

### 2.18. Statistical Analysis

The GraphPad Prism^®^ software (Version 8) was used for all analyses. Data were tested for homogeneity of variance by Bartlett’s test and for normal distribution by Shapiro-Wilk test. One-way ANOVA was performed with Tukey-Kramer multiple comparisons tests or Dunnett’s multiple comparisons tests as appropriate. Mann-Whitney tests were performed where variances were unequal. Culture data included culture replicate as a random variable. Data were presented as means ± SEM with *p* < 0.05 considered significant. The relationships between quantitative parameters (chemerin concentrations vs. sperm parameters) were investigated by Pearson’s correlation analysis.

## 3. Results

### 3.1. Chemerin and CMKLR1 mRNA and Protein in Chicken Testes during Embryo and Postnatal Development

**Figure 1:**
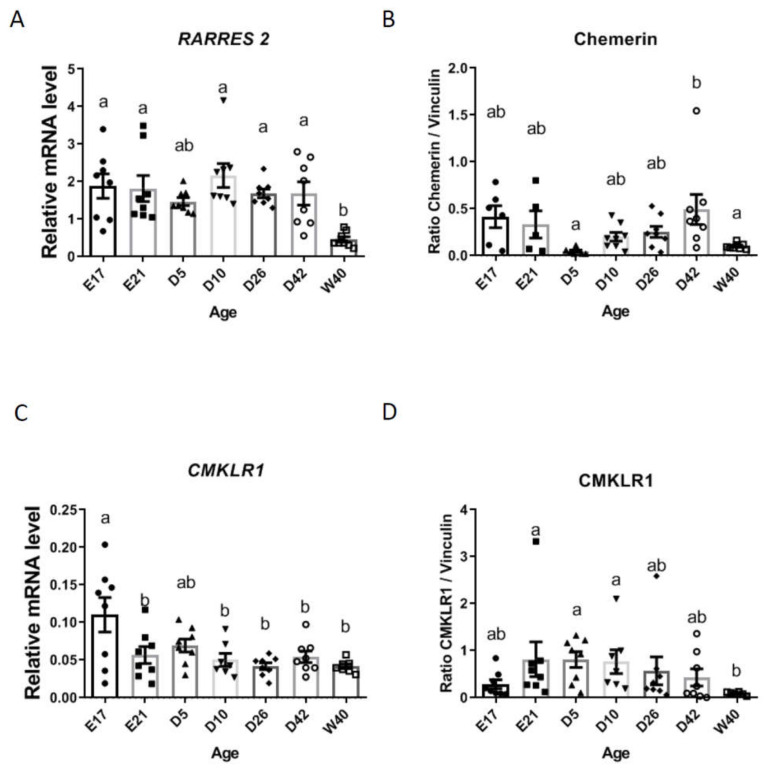
Chemerin (**A**). RARRES2 (mRNA) and (**B**). (chemerin protein)) and CMKLR1 ((**C**) (mRNA) and (**D**)(protein)) expression during embryo and postnatal development in chicken testes. Total RNA and proteins were extracted from testes of chicken at embryo day 17 (E17), hatching (E21), at 5 (D5), 10 (D10), 26 (D26), and 42 (D42)-day-old and finally at 40-week-old (W40). Gene expression and protein production were assessed by RT-qPCR and Western blotting, respectively. Data are means ± SEM of 10 testes from 10 different animals per stage. Different letters indicate a significant difference at *p* < 0.05.

As shown in Figure 1A, RARRES2 gene expression was observed at every age studied with a lower expression during adulthood (W40, 40-week-old). This result was confirmed in terms of protein by using chicken specific homemade antibodies, with a minimum of protein detection at W40 but also at D5 (Figure 1B). Similar results were shown for CMKLR1 (gene and protein) with a minimal expression at W40 (Figure 1C,D).

**Figure 2:**
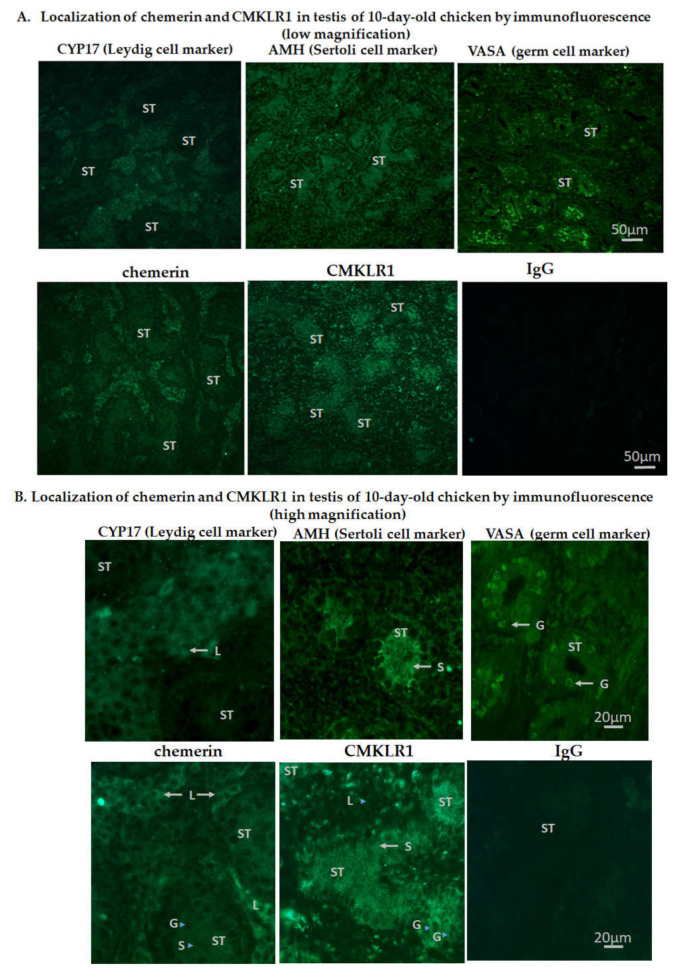
Localization of chemerin and its receptor, CMKLR1 in the testis of 10-day-old chicken by immunofluorescence. Low (**A**) scale bar = 50 µm and high (**B**) magnification scale bar = 20 µm. IgG was used as a control. Arrows indicate staining for Leydig (L), Sertoli (S), and germ (G) cells. ST: Seminiferous tubules are also shown. CYP17, AMH and VASA staining are specific for Leydig, Sertoli, and germ cells, respectively. Immunostainings are representative of 5 testicular sections from 5 different animals.

**Figure 3:**
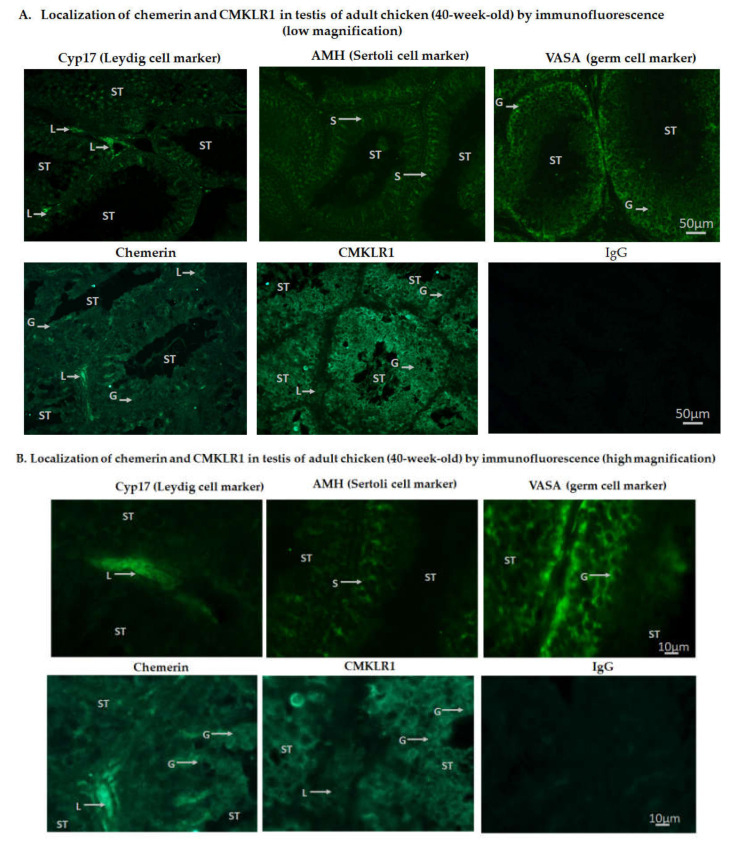
Localization of chemerin and its receptor, CMKLR1 in the testis of adult (40-week-old) chicken by immunofluorescence. Low (**A**) scale bar = 50 µm and high (**B**) magnification scale bar = 10 µm. IgG was used as a control. Arrows indicate staining for Leydig (L), Sertoli (S), and germ (G) cells. ST: Seminiferous tubules are also shown. CYP17, AMH, and VASA staining are specific for Leydig, Sertoli, and germ cells, respectively. Immunostainings are representative of 5 testicular sections from 5 different animals.

By immunofluorescence, we localized chemerin and CMKLR1 proteins in 10-day-old (Figure 2A,B) and adult (40 weeks of age, Figure 3A,B) chicken testis. In 10-day-old chicken testis, chemerin was expressed higher in Leydig cells (L) as compared to Sertoli (S) and germ (G) cells, whereas CMKLR1 was expressed higher in Sertoli cells as compared to Leydig and germ cells (Figure 2A (low magnification), B (high magnification) CYP17 (Cytochrome P450 17α Hydroxylase/17,20 Lyase), AMH (*Anti-müllerian hormone*), and VASA (also known as DDX4 (DEAD [Asp-Glu-Ala-Asp] box polypeptide 4) are specific markers for Leydig [35,36], Sertoli [29], and germ cells [37], respectively Figure 2A (low magnification), B (high magnification).

In adult chicken testis, chemerin was expressed higher in Leydig cells (L) as compared to Sertoli (S) and germ (G) cells, whereas CMKLR1 was expressed higher in germ cells as compared to Leydig cells (Figure 3A (low magnification), B (high magnification).

### 3.2. Recombinant Chicken Chemerin through CMKLR1 Inhibits hCG-Stimulated Testosterone Production by In Vitro Chicken Testis Explants

**Figure 4:**
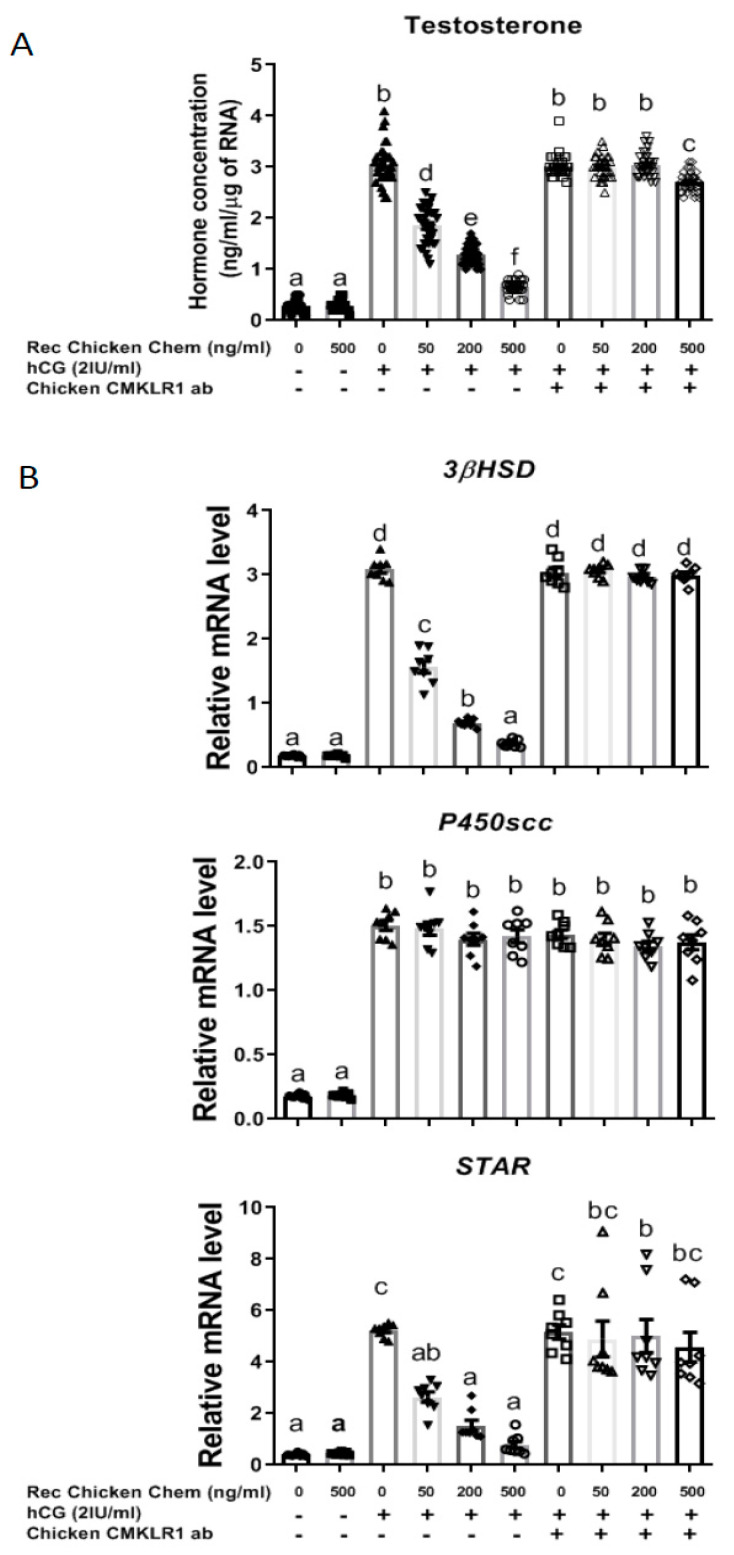
Effect of recombinant chicken chemerin (Rec Chicken Chem) on in vitro testosterone production by chicken testis explants with or without the addition of hCG and/or chicken CMKLR1 antibody. Conditioned culture media were collected, and testosterone concentrations measured by ELISA assay (**A**). Total RNA was extracted from testis explants, and gene expression was measured by RT-qPCR for 3βHSD, STAR, and P450scc as described in the materials and methods (**B**). Data are means ± SEM of 6 replicates (one replicate is representative of about two testes for each condition). Bars with different letters are significantly different (*p* < 0.05).

We then investigated the effect of chicken chemerin treatment on the production of testosterone by chicken testis explants. As shown in Figure 4A, Rec Chicken Chem did not affect basal testosterone production, whereas it reduced in a dose-dependent manner hCG-stimulated testosterone release by in vitro chicken testis explants (*p* < 0.001). Furthermore, the chicken CMKLR1 Ab totally abolished the chemerin-induced inhibition in the production of testosterone in the presence of hCG. Thus, Rec Chicken Chem strongly reduces hCG-induced testosterone production at least through CMKLR1 in chicken testis explants.

As shown in Figure 4B, Rec Chicken Chem treatment reduced in a dose-dependent manner the mRNA expression of 3βHSD and steroidogenic acute regulatory protein (STAR) in the presence of hCG, whereas no effect was observed on the mRNA levels of p450SCC. These effects were totally abolished in the presence of 10 μg chicken CMKLR1 Ab. Thus, the decrease in testosterone secretion in response to Rec Chicken Chem may be due to a reduction in the amounts of the 3βHSD and STAR expression through activation of CMKLR1.

### 3.3. Potential Signaling Pathways Involved in the Inhibitory Effect of Recombinant Chicken Chemerin on hCG-Stimulated Testosterone in Chicken Testis Explants

**Figure 5:**
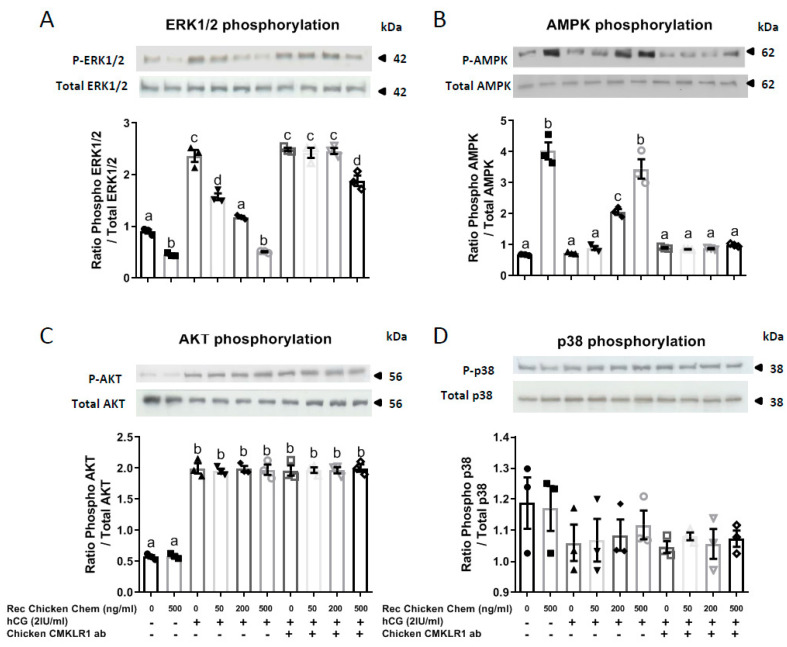
Effect of recombinant chicken chemerin (Rec Chicken Chem) on MAPK ERK1/2 (**A**), AMPK (**B**), Akt (**C**), and MAPK P38 (**D**) phosphorylation in chicken testis explants incubated with or without the addition of hCG and/or chicken CMKLR1 antibody. Testis explant lysates (50 μg) were resolved by SDS-PAGE, transferred to nitrocellulose, and probed with anti-phospho-MAPK ERK1/2 and anti-ERK1/2 (**A**), anti-phospho-AMPK and total AMPK (**B**), anti-phospho-Akt and total Akt (**C**), and anti-MAPK P38 and P38 total (**D**) antibodies. Bands on the blots were quantified, and the phosphorylated protein/total protein ratio is shown. Data are means ± SEM of 6 replicates (one replicate is representative of about two testes for each condition). Bars with different letters are significantly different (*p* < 0.05).

Thus, we first examined the ability of Rec Chicken Chem to modulate the activation of these signaling pathways, and second, determined whether these effects were dependent on CMKLR1. Quantitative analysis of Western blots indicated that Rec Chicken Chem inhibited basal and hCG-induced MAPK ERK2 phosphorylation in a dose-dependent and chicken CMKLR1 Ab totally reversed these effects (Figure 5A). Furthermore, as shown in Figure 5B, AMPK phosphorylation was significantly increased by Rec Chicken Chem, whereas it was not affected by hCG treatment (Figure 5B). Chicken CMKLR1 Ab significantly abolished the dose-dependent stimulatory effect of Rec Chicken Chem on AMPK phosphorylation (Figure 5B). At the opposite, Rec Chicken Chem and chicken CMKLR1 Ab did not affect Akt and MAPK P38 phosphorylation in basal state or in response to hCG treatment (Figure 5C,D). Thus, Rec Chicken Chem reduces hCG-induced MAPK ERK1/2 phosphorylation through the activation of CMKLR1.

### 3.4. Chemerin in Chicken Sperm: Localization of Chemerin and CMKLR1 in Chicken Spermatozoa and Correlation between Chemerin in Blood and Seminal Plasma and Semen Parameters

**Figure 6:**
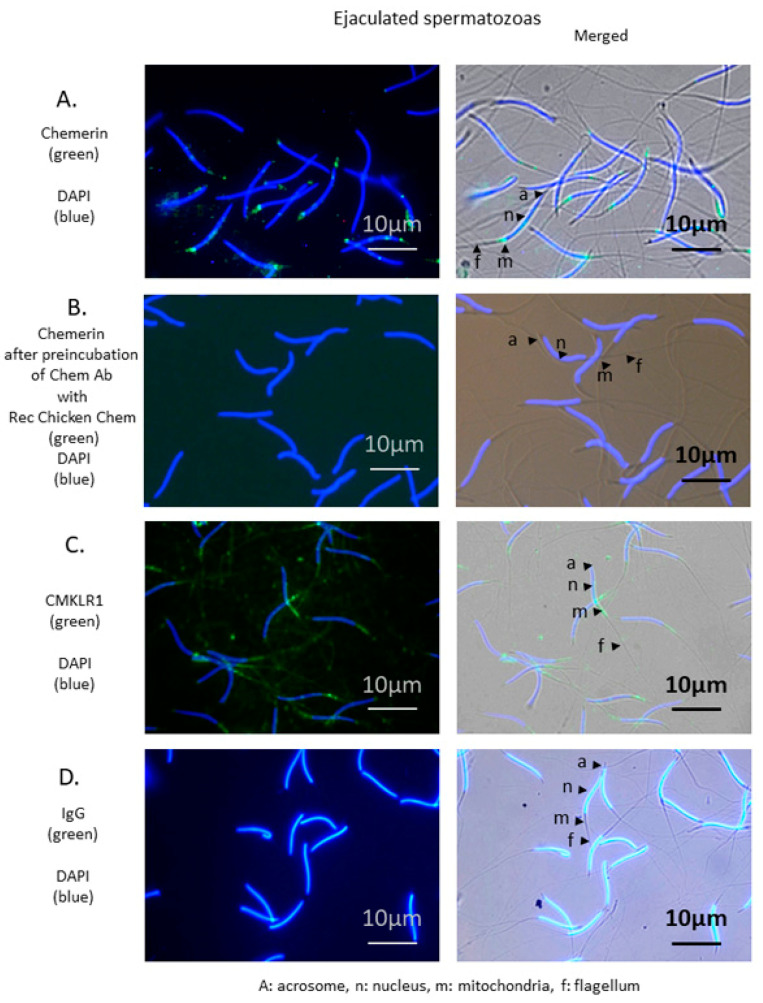
Localization of chemerin and its receptor, CMKLR1 in spermatozoa of adult roosters (40-week-old). Immunofluorescence on spermatozoa using chemerin (**A**) and CMKLR1 (**C**) antibodies shows staining of the mid-piece. Nuclei are stained in blue (DAPI), and specific antigens are stained in green. A: Acrosome, n: Nucleus, m: Mitochondria, f: Flagellum. ejaculated spermatozoa Scale bar = 10 µm. (**B**) shows the abolition of specific staining following pre-absorption of chicken chemerin antibody with the recombinant chicken chemerin. (**D**) IgG was used as a negative control. Immunostainings shown are representative of spermatozoa from three different animals.

We then examined the localization of chemerin and its receptor in the chicken spermatozoa by immunofluorescence, and we performed a correlation analysis for chicken blood and seminal plasma chemerin as determined by ELISA assay and immunoblot with semen parameters. As shown in Figure 6, strong specific staining for chemerin and CMKLR1 in the mid-piece of the spermatozoa, the zona containing mitochondria suggesting a predominant presence of the chemerin system in this particular part of the gamete.

**Figure 7:**
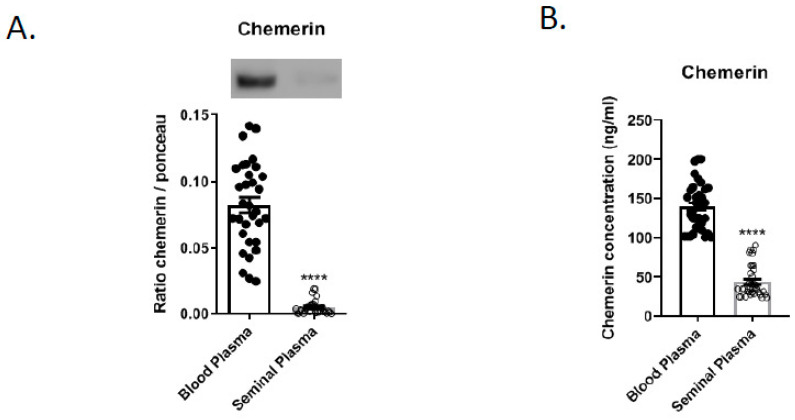
Expression of chemerin in seminal and blood plasma of adult roosters (40-week-old) by immunoblot (**A**) and ELISA assay (**B**). Data are shown as means ± SEM from 36 roosters, **** *p* < 0.001 (Student *t*-test).

Blood and seminal plasma from 36 roosters 40-weeks-old were individually evaluated. As shown in Figure 7, the chemerin amount as determined by immunoblot was ten times higher in the blood plasma (BP) compared to the seminal plasma (SP) (0.08 +/− 0.03 vs. 0.008 +/− 0.005, *n* = 36, Figure 7A). Using ELISA assay, similar results were observed; chemerin concentration was 3.3 higher in blood than seminal plasma (Figure 7B, BP 137.7 +/− 30.5 vs. 41.5 +/− 19.0).

Concentration, total and progressive motility, and static spermatozoa in sperm were measured using an Integrated Visual Optical System. After sampling, animals were killed, and testes collected and weighted. As shown in Table 1, we found negative correlations between chemerin in seminal plasma and the motile (r = −0.9331; *p* < 0.0001) and the progressive (r = −0, 8121; *p* < 0.0001) percentage of total spermatozoa and the spermatozoa concentration (r = −0.4279; *p =* 0.0092). Moreover, a positive correlation has been observed between chemerin in seminal plasma and the percentage of static spermatozoa (r = 0.9331; *p* < 0.0001). Neither blood plasma chemerin concentration nor testicular weight was correlated with seminal plasma chemerin concentration (Table 1).

### 3.5. Effects of Recombinant Chicken Chemerin on the Mass Motility of Rooster Semen

**Figure 8:**
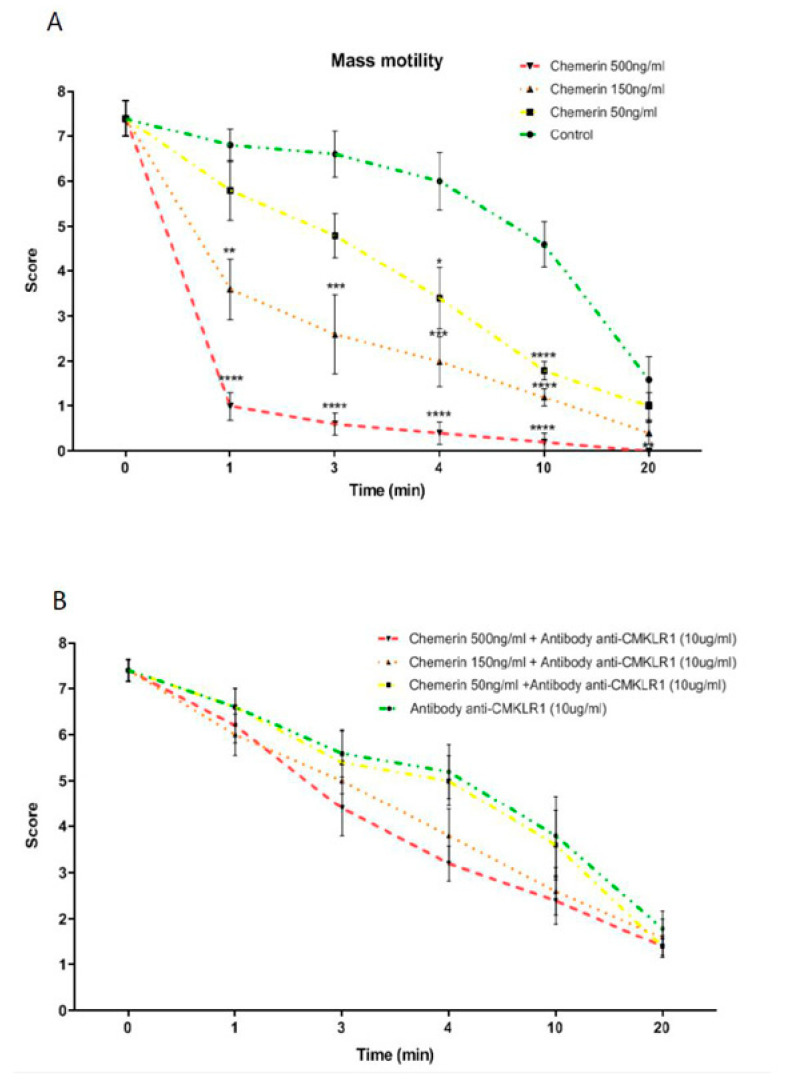
In vitro effects of recombinant chicken chemerin on mass motility of chicken spermatozoa. Sperm from adult roosters have been collected and treated with recombinant chicken chemerin at several concentrations and times without (**A**) and with (**B**) a pre-incubation of an anti-chicken CMKLR1 antibody. Data are means ± SEM of 4 replicates (4 pools of 10 roosters), and letters identify means that are significantly different (One-way ANOVA test with Tukey-Kramer multiple comparisons post-test). ** *p* < 0.05; *** *p* < 0.01 and **** *p* < 0.001.

Mass sperm motility takes into consideration the collective movement of sperm. It is associated with fertility in different species, including chicken [33,34]. Semen was collected on 5 roosters and mixed together to form one pool and then divided in 4 to constitute 4 groups (control, chemerin 50, 150, and 500 ng/mL treated group). The mass motility of semen was estimated under a confocal microscope at 1, 3, 4, 10, and 20 min. The experiment was repeated 4 times with a sperm pool from different animals. As expected, under control conditions (no stimulation), the score for mass motility decreased gradually with time. As showed in Figure 8A, we observed significant differences for chemerin treated groups with a faster decrease of the score compared to control at 4 and 10 min for the chemerin group with 50 ng/mL group and at 1, 3, 4, and 10 min for the chemerin groups with 150 and 500 ng/mL. Finally, no more mass motility was observed in the 500 ng

Chemerin group after 20 min incubation, which was significantly different from the control group (Figure 8A). We next repeated the same experiments with a pre-incubation for 30 min at 4 °C with the chicken CMKLR1 Ab (10 μg/mL). As shown in Figure 8B, this pre-treatment totally abolished the inhibitory effect of the recombinant chicken chemerin, whatever the dose and the time of stimulation suggesting that the negative effect of recombinant chicken on the mass sperm motility was mediated by CMKLR1.

### 3.6. Effects of Recombinant Chicken Chemerin on the Motility of Spermatozoa in Rooster

We next investigated the effects of various concentrations of recombinant chicken chemerin (0, 50, 150, and 500 ng/mL after 5 and 20 min of stimulation) on the individual spermatozoa motility in roosters by using Computer Assisted Sperm Analysis (CASA). As shown in Table 2, recombinant chicken chemerin at 50 and 150 ng/mL had no significant effect on the percentage of motility (MOT, %) whatever the time of stimulation, whereas chemerin at 500 ng/mL after 20 min of stimulation significantly reduced this percentage from 68 ± 1.7% (control) to 45.3 ± 5.6% (chemerin 500 ng/mL). This inhibitory effect was significantly decreased (61.2 ± 3.1%) when the sperm was pre-incubated for 30 min with the chicken CMKLR1 Ab (10 μg/mL, Table 2). We next examined whether the negative effect of recombinant chicken chemerin (500 ng/mL) on the spermatozoa motility could be responsible for an increase in the mortality level. Flux cytometry experiments showed no significant difference in the percentage of dead spermatozoa as determined by the ratio between the SYBR-14 and PI staining and in the percentage of spermatozoa with a sperm membrane intact (Table S1). Furthermore, no variation in the calcium concentration in spermatozoa and no difference in the percentage of live intact acrosomes were observed in response to recombinant chicken chemerin (Table S1), suggesting no effect of recombinant chicken chemerin on acrosomal reaction. Interestingly, we observed that chemerin at 500 ng after 20 min of stimulation significantly increased the VAP from 61.3 ± 3.7 μm/s (control) to 97.2 ± 8.7 μm/s and this effect was abolished when the sperm was pre-incubated for 30 min with the chicken CMKLR1 Ab (10 μg/mL, Table 2).

### 3.7. Effects of Recombinant Chicken Chemerin on the Egg Fertilization

**Figure 9:**
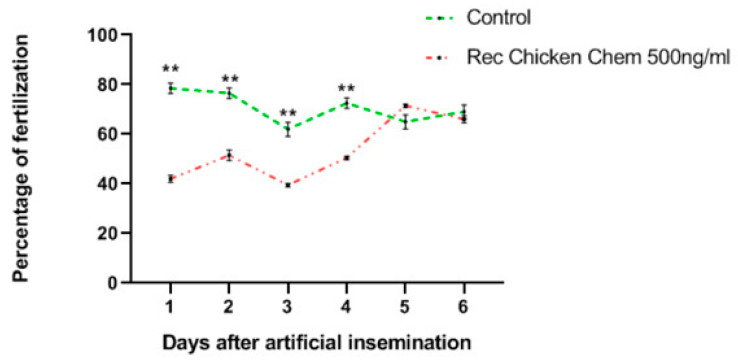
In vivo effects of recombinant chicken chemerin (Rec Chicken Chem) on chicken sperm fertility. The graph shows the fertility rate after eggs candling at day 7. Data are percentages of fertilized eggs per group and per day of collection and asterisks identify means that are significantly different from the control (vehicle group) at ** *p* < 0.01 (Student *t*-test).

We next determined whether the negative effect of recombinant chicken chemerin on the quality of rooster spermatozoa could affect the in vivo egg fertilization level. The percentage of fertilized eggs after 7 days of incubation is shown on Figure 9 and similar data were obtained after 14 days of incubation and at hatching. Results show that eggs laid by hens inseminated with sperm treated with chemerin 500 ng/mL exhibit a fertility rate significantly lower than the control group for the first 4 days of egg pickup after artificial insemination. However, from the day 5 to day 6 of egg collection, the fertility rates were similar between the two groups (Figure 9).

## 4. Discussion

**Figure 10:**
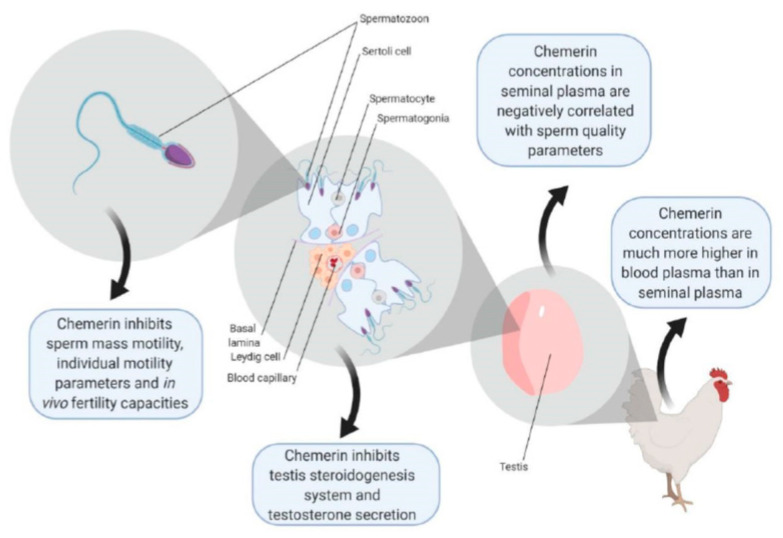
A summary of chicken chemerin effect on testis function and fertility in broiler breeder chicken.

By using specific chicken chemerin system tools (recombinant chemerin protein, chemerin, and CMKLR1 antibodies and ELISA assay), we showed that chemerin and its receptor, CMKLR1, are present in testicular cells and sperm of roosters. Furthermore, chemerin through CMKLR1 inhibited in vitro hCG stimulated testosterone production, and this was associated with a reduction in 3βHSD and STAR expression and MAPK ERK1/2 phosphorylation (Figure 10). We also reported that chemerin concentration was lower in seminal plasma than in blood plasma. Moreover, chemerin seminal plasma concentration was in vivo negatively correlated with spermatozoa concentration and motility (Figure 10). Furthermore, we demonstrated that recombinant chicken chemerin through CMKLR1 inhibited in vitro mass and individual motility of spermatozoa. These data might explain a lower in vivo fertilization of eggs when hens are in vivo artificially inseminated with spermatozoa incubated with chemerin. Our study is the first to report the expression of chemerin and its receptors CMKLR1 in rooster testis and spermatozoa and to identify its direct biological effects in avian male gonad and sperm (Figure 10).

In chicken, we observed that chemerin and CMKLR1 gene expression was lower in adulthood than in embryo or during the neonatal period. Concerning chemerin expression, our result is consistent with the data obtained in the rat by Li et al., which showed a decrease from the neonatal period to adulthood [10]. However, in this same study, they observed an increase in CMKLR1 expression, suggesting a species-dependent regulation for this receptor. Chemerin is able to bind two other receptors, called GPR1 and CCRL2 [7]. Their expression remains to be investigated in chicken testis. In chicken testis, we showed that chemerin and its receptor are expressed in Leydig and Sertoli cells and germinal cells. We will discuss the potential role of chemerin and CMKLR1 in Leydig cells and spermatozoa below according to our results. Sertoli cells play a crucial role in the testis since they support spermatogenesis as a nurse cell [38]. In highly purified adult human Sertoli cells, a recent study showed that high chemerin concentration significantly reduced FSH receptor expression and could negatively affect Sertoli cell maturation and retain them in a more prepubertal stage [39]. The effect of chemerin on chicken Sertoli remains to be determined. Like in mammals, the quantity and quality of germ cells produced in a given male are dependent on the number and function of Sertoli cells [40]. We found that CMKLR1 is present in chicken spermatogonia. These data are in good agreement with those observed in rats [10].

In the present study, we showed that chicken chemerin inhibits not basal, but hCG-stimulated testosterone production by testis explants, and this was associated with a reduction in 3βHSD and STAR expression and MAPK ERK1/2 phosphorylation. We confirmed the absence of observing a band at ∼44 kDa corresponding to MAPK ERK1 in the chicken, as already described in many studies [41,42]. Testosterone is the male steroid hormone that stimulates differentiation of the male phenotype and spermatogenesis in the testes. A negative effect of chemerin has also been described in rat Leydig cells [10]. In the Leydig cell, after stimulation by luteinizing hormone (LH), cholesterol is mobilized to the inner mitochondrial membrane by STAR [43], where it is converted to pregnenolone, by cytochrome P450 side-chain cleavage enzyme (CYP11A1, encoded by Cyp11a1). Pregnenolone then diffuses out of the mitochondrion into the membranes of the smooth endoplasmic reticulum, which contains 3β-hydroxysteroid dehydrogenase 1 (3βHSD) involved in the conversion of pregnenolone into progesterone. Progesterone is then sequentially converted to 17-hydroxyprogesterone, androstenedione, and, ultimately, to testosterone. Thus, chemerin could reduce testosterone production in response to hCG in chicken Leydig cells by inhibiting STAR and 3βHSD expression. In rat Leydig cells, chemerin inhibits steroidogenesis through only 3βHSD downregulation [10]. Similar data were obtained in response to C20, a chemerin-Derived Peptide [11]. However, in the explants of testes from 10-day-old chicken, we observed that chemerin inhibits not only 3βHSD but also STAR suggesting a species-specific regulation of STAR by chemerin. Transcriptional regulation of the STAR gene is complex [44]. Hormone-stimulated STAR transcription may be regulated by either direct activation of transcription factors already bound to the promoter, or recruitment of other transcription factors and co-activators, or through the release of DAX-1 repression, or a combination of these mechanisms [45]. Several consensus binding sites within its promoter have been identified and characterized. The promoter of the STAR gene contains elements involved in its positive and negative regulation. We can hypothesize that these elements are differently regulated by chicken and rodent chemerin in response to hCG.

In the ovary, chemerin also inhibits steroidogenesis (estradiol or/and progesterone) in granulosa cells from various species, including humans [46], bovine [12], and rodent [47] and these effects are also associated with the inhibition of expression of steroidogenesis components. Interestingly we showed that the chicken CMKLR1 antibody abolished the inhibitory effects on the testosterone production. In bovine granulosa cells, the negative effects of chemerin on steroidogenesis are also dependent on CMKLR1 [12]. These data are in good agreement with our results, suggesting that chemerin through CMKLR1 inhibits testosterone production by chicken Leydig cells. In the rodent, some evidence shows that CMKLR1 may act to promote testosterone secretion through its another ligand named resolvin E1 [48,49]. Thus, in chicken, it remains to determine whether in vivo, resolving E1 is involved in the negative effect of CMKLR1 in testosterone production. Chemerin is known to modulate various signaling pathways. In chicken testis explants, we showed that chemerin reduced hCG stimulated MAPK ERK1/2 phosphorylation and this is abolished by using chicken CMKLR1 ab. The involvement of the MEK/MAPK ERK pathway as a mediator of the actions of LH or hCG on testosterone production by Leydig cells has been demonstrated in several in vitro studies [50,51] but also in vivo [52]. In the present study, we can hypothesize that chemerin may reduce the levels of cAMP induced by hCG. Cross-talk between CMKLR1 and LH could also be considered. Several transcription factors (e.g., NURR-1, NGFI-B, NR5a1 (previously known as steroidogenic factor-1) and NR5a2 (also known as liver receptor homolog-1)) are involved in the modulation of gene expression of steroidogenic enzymes via multiple signaling pathways including MAPK ERK1/2. The transcription factor SF-1 not only regulates STAR protein but may also modulate many other steroidogenic genes, including 3βHSD [53]. In the ovary, the suppression of steroid production and steroidogenic enzyme expression by chemerin was achieved, at least partially, via down-regulating NR5a1/2 expression and interfering with their transcriptional activity [54]. In addition, it has been shown that STAR protein expression induced by different treatments such as bufalin or cinobufagin is dependent on the activation of ERK1/2 and the involvement of SF-1 transcription factor in the human adrenocortical cells [55]. Thus, it will be interesting to investigate further the effect of chemerin on transcription factors such NR5a2 in chicken testis explants.

In our study, we observed that AMPK phosphorylation was significantly increased by Rec Chicken Chem, whereas it was not affected by hCG. Moreover, chicken CMKLR1 Ab significantly abolished the dose-dependent stimulatory effect of Rec Chicken Chem on AMPK phosphorylation. In human granulosa cells, we previously showed that chemerin decreased MAPK ERK1/2 phosphorylation, whereas it increased those of AMPK [46]. In both murine Leydig tumor cell lines (MA-10 and mLTC-1 cells), activation of AMPK inhibits cAMP-induced steroidogenesis by repressing the expression of key regulators of steroidogenesis such as STAR [16]. Thus, in our present study, in the basal state (without hCG stimulation), the activation of AMPK and the inhibition of MAPK ERK1/2 in response to chemerin treatment could inhibit testosterone production in chicken Leydig cells. Akt and MAPK P38 signaling pathways were not modulated in response to chemerin in the basal state or in response to hCG, suggesting that these signaling pathways were not involved in steroid production or other functions of this hormone in chicken testis. In rat Leydig cells, LH or hCG has been shown to stimulate IGF-I (insulin-like growth factor I) secretion and also to upregulate IGF-I receptor gene expression [56,57,58]. IGF-I has been shown to regulate Leydig cell steroidogenesis in several species [59]. In a previous study in human ovarian cells, we showed that chemerin reduces IGF-1-induced steroidogenesis and cell proliferation through a decrease in the activation of IGF-1R signaling pathways [46]. Our chicken testis explant experiment was performed in DMEM medium without serum, thus there was no exogenous IGF1. However, if chicken testicular cells, including Leydig cells, are able to produce and release IGF-1 like in mammals’ testicular cells, we could hypothesize that the negative effect of chemerin on hCG-induced testosterone could be due to alteration of IGF-1R signaling. Thus, it will be interesting to investigate the effects of chemerin on the amount of IGF-1R, beta subunit tyrosine phosphorylation in chicken testis explants in the presence and in the absence of hCG.

In our study, we observed that chemerin concentration in seminal plasma was ten (as determined by Western-blot) or three (as determined by ELISA assay) times lower than in blood plasma in the rooster. The difference between the method (Western-blot and ELISA assay) could be explained by the sensitivity of the techniques. In humans, Thomas et al., demonstrated that chemerin concentrations were four times higher in blood plasma than seminal plasma in men [21]. Thus, our data obtained in the chicken are in good agreement with those observed in humans. However, it is important to note that the origin of seminal plasma is different in birds and in most mammals.

In domestic chickens, the seminal fluid of ejaculates consists of two components: Seminal plasma and transparent fluid. Seminal plasma is produced by the testis and vas deferens, and sperm are expected to be continuously exposed to these secretions as they leave the epididymis [60]. Transparent fluid, a plasma-like secretion produced from the tumescent lymphatic folds surrounding the cloaca, has a similar composition to a dialysate of blood plasma, and is mixed with sperm upon ejaculation [61]. In the present study, we demonstrated that chemerin seminal plasma concentration but not chemerin blood plasma was negatively correlated with spermatozoa concentration and motility in vivo in chicken sperm. In human, Bobjer et al., demonstrated that chemerin concentration in serum was negatively correlated with male fertility parameters such as plasma LH, E2, and SHBG in men [22]. They also found that sub-fertile men exhibited significantly lower chemerin blood plasma concentrations as compared to fertile men. Thomas et al., in 2013, observed that chemerin concentration in seminal plasma was positively correlated with spermatozoa concentration in sperm of men, whereas it was negatively correlated with the total motility of spermatozoa [21]. In our study, we showed that chemerin through CMKLR1 inhibited in vitro mass and individual spermatozoa motility in chicken whereas it did not affect the viability of spermatozoa. Moreover, the integrity of the acrosome membrane was intact suggesting that chemerin did not alter the acrosome reaction. In humans, MAPK ERK1/2 is considered as a regulator of sperm motility and a predictor of human sperm quality [62]. Thus, it will be interesting to determine the molecular mechanism involved in the inhibitory effect of chemerin on chicken spermatozoa motility. Interestingly we showed that chicken sperm treated by chemerin reduced egg fertility during the four first days after artificial insemination. In birds, like other species, in which copulation is consistently asynchronous with ovulation, spermatozoa after artificial insemination are stored in the special structure of sperm storage tubules (SSTs) in the oviduct of females [63]. The ability to store sperm in the hens can directly affect the fertilization rate of hatching eggs, which is an important reproductive strategy in the laying hens [63]. SSTs microenvironment and regulation of sperm metabolism by SST cells are vital factors for prolonged sperm storage [64]. SSTs supply nutrients to the sperm and remove any waste products of sperm metabolism [65]. Thus, we can speculate that after 4 days in SSTs chemerin was degraded or its negative effects on the sperm were neutralized.

## 5. Conclusions

The summary diagram shows the effects of chicken chemerin on the chicken testis and male fertility reported in this paper (Figure 10). Our results supply direct evidence that chemerin through CMKLR1 negatively regulates in vitro chicken testosterone production, and this is associated with a reduction in 3βHSD and STAR expression and MAPK ERK2 phosphorylation. Furthermore, we provide evidence that chemerin seminal plasma concentration is negatively correlated with sperm quality (spermatozoa concentration and motility). Chemerin also inhibits mass and individual motility of spermatozoa in chicken, and this could explain a lower in vivo fertility of eggs. Further investigations are necessary to understand the molecular mechanisms involved in the failure of fertilization when chicken spermatozoa are treated with chemerin.

## Figures and Tables

**Table 1:** Correlations between chemerin concentrations in blood and seminal plasma and sperm quality parameters. Individual qualitative sperm parameters from 36 roosters 40-week-old were measured by Computer Assisted Sperm Analyzer (CASA). Pearson’s correlation analysis is presented below. Asterisks and red text identify correlations that are significantly different.

	Motile Spermatozoa (%)	Progressive Spermatozoa (%)	Static Spermatozoa (%)	Concentration Spermatozoa (×10^9^)/ml	Weight Testis (g)	Chemerin Concentration Blood Plasma (ng/mL)	Chemerin Concentration Seminal Plasma (ng/mL)
Chemerin concentration blood plasma (ng/mL)	r = 0.0408 *p* = 0.8134	r = 0.0689 *p* = 0.6895	r = −0.0408 *p* = 0.8134	r = −0.0882 *p* = 0.6091	r = 0.3173 *p* = 0.0675	-	-
Chemerin concentration seminal plasma (ng/mL)	r = −0.9331 **** *p* < 0.0001	r = −0.8121 **** *p* < 0.0001	r = 0.9331 **** *p* < 0.0001	r = −0.4279 ** *p* = 0.0092	r = −0.1007 *p* = 0.5710	r = 0.04945 *p* = 0.7746	-

**Table 2:** Effect of chicken recombinant chemerin on sperm motility in fresh chicken semen. Sperm samples from individual adult roosters were treated with recombinant chicken chemerin and with/without chicken CMKLR1 antibody (CMKLR1 Ab) pretreatment. Motility parameters were assessed by using Computer Assisted Sperm Analysis (CASA). They were the percentage of motile sperm (MOT); average path velocity (VAP); straight-line velocity (VSL); curvilinear velocity (VCL). Data are represented as mean ± SEM (*n* = 36 experiments using one sample of semen per adult rooster).

Parameter	Time	Control	Chemerin 50 ng/mL	Chemerin 150 ng/mL	Chemerin 500 ng/mL	Chicken CMKLR1 Ab (10 mg/mL)	Chicken CMKLR1 Ab (10 μg/mL) + Chemerin 500 ng/mL
**MOT (%)**	T = 5 min	74.03 ± 4.59	69.17 ± 4.87	70.00 ± 5.63	72.17 ± 3.90	73.83 ± 3.77	76.83 ± 2.71
	T = 20 min	68.00 ± 1.69	64.67 ± 1.28	63.33 ± 2.62	45.33 ± 5.62 ** (*p* = 0.0003)	59.67 ± 2.74	61.17 ± 3.11
**VAP (mm/s)**	T = 5 min	74.18 ± 7.20	76.44 ± 4.92	81.34 ± 9.24	77.08 ± 8.35	80.42 ± 8.87	78.98 ± 6.65
	T = 20 min	61.26 ± 3.71	76.32 ± 5.28	76.62 ± 9.17	97.62 ± 8.70 * (*p* = 0.016)	72.46 ± 7.83	68.06 ± 6.74
**VSL (mm/s)**	T = 5 min	54.97 ± 5.72	58.22 ± 4.23	60.53 ± 5.44	56.32 ± 5.82	59.62 ± 6.21	59.38 ± 5.52
	T = 20 min	47.70 ± 5.48	60.72 ± 6.18	59.28 ± 7.32	70.75 ± 4.25	58.15 ± 7.95	54.70 ± 7.17
**VCL (mm/s)**	T = 5 min	138.40 ± 7.30	140.80 ± 4.35	145.40 ± 10.35	142.10 ± 8.88	146.20 ± 9.96	145.40 ± 6.28
	T = 20 min	121.90 ± 5.95	149.6 ± 9.78	144.30 ± 10.68	155.10 ± 7.19	140.30 ± 11.69	129.90 ± 8.72

## Supplementary Materials

The following are available online at https://www.mdpi.com/2073-4409/9/7/1599/s1, Table S1: Effect of chicken recombinant chemerin on spermatozoa membrane and acrosome integrity and intracellular calcium concentration in spermatozoa. Data are means ± SEM. The experiment was performed at two times (5 and 20 min) after different treatments (chemerin 50 ng/mL, chemerin 150 ng/mL, chemerin 500 ng/mL, chicken CMKLR1 Ab (10 mg/mL) and chicken CMKLR1 Ab (10 μg/mL) + chemerin 500 ng/mL). It was repeated six times using each time different pool of sperm from 10 roosters). No significant difference was observed for all the parameters at *p* < 0.05. Figure S1: Characterization of the chicken CMKLR1 antibody (CMKLR1 ab). (A) Protein sequence alignment of human (NM_004072), and chicken (NM_001282407) CMKLR1 using the Database Resources of the National Center for Biotechnology Information. The box represents the sequence of two peptides used to produce chicken CMKLR1 ab. Detection of CMKLR1 (42 kDa) in adult chicken (1–3) and human (4–5) tissues by immunoblotting using the chicken CMKLR1 antibody or pre-immune serum. Chicken abdominal adipose tissue (1); chicken Liver (2); chicken pectoralis major muscle (3); human subcutaneous adipose tissue (4); human granulosa cells (5). Human lysates were obtained, as described in Reverchon et al. [46].
