# Supplementary material for: Chemerin Impairs In Vitro Testosterone Production, Sperm Motility, and Fertility in Chicken: Possible Involvement of Its Receptor CMKLR1
Source: Cells. 2020 Jul 1;9(7):1599. doi: 10.3390/cells9071599 (PMC7408590; doi:10.3390/cells9071599)

A.

```
Chicken   1 MALSNLSEYSDDSDTY-DYLDYTYEEPGSVWTGPSYDPKD--VARILSVVIYSVSCVLGI  57
Human     1 MEDEDYNTSISYGDEYPDYLD-------SIVVLEDLSPLEARVTRIFLVVVYSIVCFLGI  53

Chicken  58 LGNGLVIAIITLKMKKSVNAIWFLNLAVADFLFNIFLPINIAYTAMRYNWIFGTVMCKLN  117
Human    54 LGNGLVIIIATFKMKKTVNMVWFLNLAVADFLFNVFLPIHITYAAMDYHWVFGTAMCKIS  113

Chicken 118 SFLLILNMYTSVLLLTTISFDRYVSVVFPVWSQNHRSTNLACMVCLIIWMVGIIMSCPSL  177
Human   114 NFLLIHNMFTSVFLLTIISSDRCISVLLPVWSQNHRSVRLAYMACMVIWVLAFFLSSPSL  173

Chicken 178 VFRDTAQARNSIICFSNFSLSRNRS--------YQALALVRHRTVNITRLFAGFLLPITI  229
Human   174 VFRDTANLHGKISCFNNFSLSTPGSSSWPTHSQMDPVGYSRHMVVTVTRFLCGFLVPVLI  233

Chicken 230 ITFCYISIALNLRRNRLAKSKKPFKIIVTIIVTFFLCWSPYHLLNILETEPDLTPRSVFE  289
Human   234 ITACYLTIVCKLQRNRLAKTKKPFKIIVTIIITFFLCWCPYHTLNLLELHHTAMPGSVFS  293

Chicken 290 IGVPITTALAASNSCMNPVLYVFMGQDFKKFKVTILSRLVNALSEETGHSSIV-HRSFSK  348
Human   294 LGLPLATALAIANSCMNPILYVFMGQDFKKFKVALFSRLVNALSEDTGHSSYPSHRSFTK  353

Chicken 349 MSSMTEK------ETTVL  360
Human   354 MSSMNERTSMNERETGML  371
```

B.

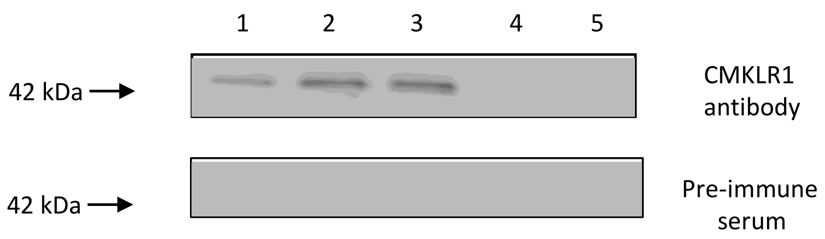

Supplement: Supplementary file 1 [file cells-09-01599-s001.zip › Figure S1.pdf]
