# Supplementary material for: Chemerin Impairs In Vitro Testosterone Production, Sperm Motility, and Fertility in Chicken: Possible Involvement of Its Receptor CMKLR1
Source: Cells. 2020 Jul 1;9(7):1599. doi: 10.3390/cells9071599 (PMC7408590; doi:10.3390/cells9071599)
Supplement: Supplementary file 1 [file cells-09-01599-s001.zip › Table S1.pdf]

Parameter	Time	Control	Chemerin 50 ng/ml	Chemerin 150 ng/ml	Chemerin 500 ng/ml	Chicken CMKLR1 Ab (10 μg/ml)	Chicken CMKLR1 Ab (10 μg/ml) + Chemerin 500 ng/ml
Dead (SYBR14/PI staining) (%)	T = 5 min	9.16 ± 0.72	11.07 ± 3.35	11.37 ± 4.28	12.10 ± 2.60	10.56 ± 2.28	11.24 ± 1.89
	T = 20 min	14.68 ± 4.37	15.58 ± 3.61	14.16 ± 3.66	14.98 ± 3.97	15.12 ± 5.28	16.76 ± 4.54
Live intact acrosomes (%)	T = 5 min	82.87 ± 4.48	81.56 ± 4.34	80.76 ± 4.58	78.94 ± 4.95	81.03 ± 4.95	79.96 ± 3.92
	T = 20 min	77.00 ± 4.99	75.41 ± 5.37	77.77 ± 4.55	75.23 ± 5.40	74.83 ± 5.74	73.39 ± 6.07
Ca^{2+} (μg/ml)	T = 5 min	34.52 ± 0.45	34.05 ± 1.16	36.83 ± 3.38	33.60 ± 0.77	34.68 ± 0.43	33.29 ± 0.48
	T = 20 min	37.54 ± 3.51	34.41 ± 0.71	34.09 ± 0.82	34.41 ± 0.79	35.39 ± 1.48	34.41 ± 1.48
